# Effect of health system reforms in Turkey on user satisfaction

**DOI:** 10.7189/jogh.05.020403

**Published:** 2015-12

**Authors:** Jonathan Stokes, Ipek Gurol–Urganci, Thomas Hone, Rifat Atun

**Affiliations:** 1 NIHR Greater Manchester Primary Care Patient Safety Translational Research Centre, Institute of Population Health, University of Manchester, Manchester, UK; 2 Department of Health Services Research and Policy, London School of Hygiene and Tropical Medicine, London, UK; 3 Department of Primary Care and Public Health, Imperial College London, London, UK; 4 Harvard T.H. Chan School of Public Health, Harvard University, USA

## Abstract

In 2003, the Turkish government introduced major health system changes, the Health Transformation Programme (HTP), to achieve universal health coverage (UHC). The HTP leveraged changes in all parts of the health system, organization, financing, resource management and service delivery, with a new family medicine model introducing primary care at the heart of the system. This article examines the effect of these health system changes on user satisfaction, a key goal of a responsive health system. Utilizing the results of a nationally representative yearly survey introduced at the baseline of the health system transformation, multivariate logistic regression analysis is used to examine the yearly effect on satisfaction with health services. During the 9–year period analyzed (2004–2012), there was a nearly 20% rise in reported health service use, coinciding with increased access, measured by insurance coverage. Controlling for factors known to contribute to user satisfaction in the literature, there is a significant (*P* < 0.001) increase in user satisfaction with health services in almost every year (bar 2006) from the baseline measure, with the odds of being satisfied with health services in 2012, 2.56 (95% confidence interval (CI) of 2.01–3.24) times that in 2004, having peaked at 3.58 (95% CI 2.82–4.55) times the baseline odds in 2011. Additionally, those who used public primary care services were slightly, but significantly (*P* < 0.05) more satisfied than those who used any other services, and increasingly patients are choosing primary care services rather than secondary care services as the provider of first contact. A number of quality indicators can probably help account for the increased satisfaction with public primary care services, and the increase in seeking first–contact with these providers. The implementation of primary care focused UHC as part of the HTP has improved user satisfaction in Turkey.

Starting in 2003, the Turkish government introduced major health system reforms to achieve universal health coverage (UHC) [1], the Health Transformation Programme (HTP), led by the Ministry of Health (MoH) with collaboration of international agencies such as the World Health Organization (WHO) [2].

The HTP brought changes to organization, financing, resource management and service delivery in the Turkish health system to address large inequities in health insurance coverage. In 2003, only 66.3% of the population was covered by health insurance. However, just 12% of the poorest expenditure decile benefited from the Green Card scheme (a noncontributory health financing scheme for the poor, separate from the health insurance schemes, financed by the Ministry of Finance and operated by the Ministry of Health, covering until 2004 cost of hospital inpatient care, but not outpatients or medicines) [1], which in 2003 covered 2.5 million people [3].

The population which lacked health insurance experienced high out–of–pocket expenditures, had variable access to health services and experienced poor health outcomes [1]. However, absolute shortage and inequitable distribution of physical infrastructure and health human resources meant that even for the insured, access to health services proved challenging. Furthermore, the dual practice of doctors in teaching hospitals (where doctors practiced both in the private and public sectors) substantially reduced the availability of public services for the insured, with many patients diverted to private practice. In particular, for financing, the health system reforms included the extension of health insurance to almost 11 million persons funded from government budget.

The health system reforms of financing were aimed at consolidating into a general health insurance organization and aligning the five parallel social health insurance schemes, namely: the Social Insurance Organization (SIO) (covering active and retired workers from the formal sector); Government Employees Retirement Fund (covering retired civil servants); Bağ–Kur (covering the self–employed and artisans); the Active Civil Servants Insurance Fund (covering civil servants in work and their dependents); and, the Green Card scheme (for poor households with incomes below the national minimum). Each of these schemes had different benefit packages and disparate contractual arrangements with provider organizations, leading to significant inefficiency and inequity within the health system. In addition, there existed a small but growing private sector with its own system of private insurers and health care providers [1].

Major changes in service delivery included the introduction and expansion of a new Family Medicine (FM) model, aimed at transforming countrywide the delivery of Primary Health Care (PHC) services, especially in rural areas. Introduction of provider choice in 2004 enabled patients to switch health service providers [4]. **Box 1** shows a time–line of the key financing and service delivery changes relating to patient experience.

Box 1 **Timeline of Health Transformation Programme implementation**

**2003:** Ambulance services made free; Patients no longer permitted to be held in hospitals for non–payment of fees [5].

**2004:** Green Card insurance (social security scheme for the most disadvantaged) holders covered for outpatient care and pharmaceuticals [1,5]; Conditional cash transfers introduced for pregnant women and children from most deprived households (covering 6% of population) to encourage use of services [1]; Major changes in pharmaceutical policy leads to reduction in price of drugs [1]; Patient’s Right to choose a physician implemented in Ministry of Health hospitals [5]; electronic system for complaints and suggestions introduced; and Patient choice of health care provider (secondary/primary care; public and private) introduced [1].

**2005:** New family medicine model introduced in Düzce province [5].

**2006:** Family medicine introduced in Adıyaman, Denizli, Edirne, Eskişehir, and Gümüşhane provinces [5].

**2007:** Free at delivery primary care introduced for all, regardless of insurance status; Family medicine model introduced in Elazığ, Isparta, Izmir and Samsun provinces [5].

**2008:** Free emergency and intensive care services to be provided for everyone at private as well as public hospitals [1]; Air ambulance introduced, free–of–charge to entire population [1]; Cost–sharing for complex conditions in private hospitals scrapped [1].

**2009:** Mobile pharmacy introduced to rural regions [1]; Hospital appointment system centralised [1]; Shared payment for outpatient physician and dental services introduced [5]; Family medicine introduced to five provinces (Bursa, Rize, Trabzon, Tunceli and Uşak) [5].

**2010:** Family medicine model implemented nationwide [1,5].

Collectively, these changes, amongst others, enabled the development of a unified health insurance system and to expand health care access to establish UHC by 2011 [1,6]. The health system reforms were designed to improve the user experience of the health system, which in 2003 was the lowest among the five major public services (health services, security, pensions, social security, and judiciary) – only 30% of the population were satisfied with the health service, where the satisfaction for the other services ranged from 50–75% [1].

User satisfaction is one of the key goals of a health system, as recognized in health system frameworks [1,7]. For the purpose of this study, satisfaction is defined as “the feeling arising from meeting the needs and desires” of the individual: a definition is taken from the Life Satisfaction Survey (LSS) in Turkey which provides the data analyzed in this paper [8]. Clearly evident in the definition is the subjectivity of the concept of “satisfaction”. Being a subjective concept, a large number of factors are found to influence satisfaction at the individual level. Health system design, how care is delivered and individual characteristics influence user satisfaction with health services [9]. **Table 1** shows a summary of these factors identified in the literature.

**Table 1:** Summary of individual and systematic factors influencing user satisfaction with health services

Individual characteristics	System characteristics
**Age:** Older people are generally more satisfied [10-15] **Gender:** Some studies showing females are generally more satisfied, [11,16] although some showing more inconsistent results with the direction of effect [10,12,13,15] **Education level:** less educated are found to be more satisfied in some studies [16,17], inconsistent direction in others [12,13] **Geographical variation:** rural areas tend to be more satisfied than urban population [10] **Health status:** people in a good state of health tend to be more satisfied, [13,14] but findings are somewhat inconsistent [10,17-19] **Frequency of visits to doctor:** increased frequency, increase in satisfaction [20] **Psycho–social determinants:** various determinants associated, [10,21] most importantly seems to be ‘prior expectations of the patient’ [22]. Lower expectations, higher satisfaction [19] **Outcome satisfaction:** increased satisfaction with better health outcomes [10,14,19,21] **Socioeconomic status:** inconsistent direction depending on variable used [18] **Ethnicity:** little consistency [12,13]	**Physician level:** patient–centeredness and professional skills positively affect satisfaction [10,11,14,15,17,18,20,21,23-26] **Visible facilities:** more visibly pleasing, clean etc. facilities associated with increased satisfaction [24] **Accessibility:** Cost, availability, convenience of care, and waiting times all have effects on satisfaction [10,12,16,23,24,27] **Choice of provider:** less choice associated with less satisfaction [16,23] **Continuity of care:** more continuous care increases satisfaction [10,18,27] **Completeness of care:** more complete care offered by physicians increases satisfaction [27] **Service delivery:** more efficient processes, organized procedures, and quality of services increase satisfaction [19,26-28]

To date few studies have analyzed user satisfaction with a nationally representative sample through a period of health system reforms (**Table 1**). This study uses a nationally representative annual population with a baseline at the start of the health reforms in Turkey. The data for nine consecutive years (2004–2012) of a nationally representative population surveys undertaken annually, coinciding with the time–period of the introduction of major health system reforms in Turkey, and uses satisfaction with other public services as comparators to health services, all unique in the breadth of current literature relating to user satisfaction. In this study we adjust for individual characteristics (personal, demographic and socio–economic characteristics of the respondents) to show the effects related to key system characteristics, which have changed with the rollout of HTP, have had on user satisfaction with health services in Turkey.

## METHODS

### Data and variables

The LSS in Turkey was implemented in 2003 as part of the Urgent Action Plan of the new Government. This plan included a duty to measure the satisfaction and expectations of citizens in all areas of the country. The LSS was first carried out as part of the Household Budget Survey, but from 2004 onwards was carried out separately on an annual basis [8], with questions fairly consistent and comparable across the years [29]. These questions are based on previous surveys and are a validated instrument for measuring satisfaction [30].

The LSS uses a two–stage stratified cluster (with household as the cluster unit, and all members of the household over 18 years of age interviewed) sampling technique, with questionnaires filled in via face–to–face interviews using laptop computers. In the first stage, the sample is selected from clusters made up of an average of 100 households. The second stage uses address sampling to systematically determine the selection from this sample. Using this technique, all localities within Turkey’s borders and all citizens over the age of 18 are represented (excluding ‘institutional populations’ eg, those in hospitals, hotels, army barracks etc.) [31].

As well as health–specific questions, a number of demographic details and satisfaction with other public services are recorded from respondents meaning these can be controlled for at the individual–level when analyzing the data. **Table 2** shows the number of respondents each year of the survey, with a total sample of n = 62 933 in the nine annual surveys undertaken between 2003 and 2012 that coincide with the health system reforms.

**Table 2:** Sample size of Life Satisfaction Survey, by year

Year	Sample size of the survey
2004	6714
2005	6983
2006	6432
2007	6442
2008	6465
2009	7546
2010	7027
2011	7368
2012	7956

The question used as the outcome measure for the analyses was: “Satisfaction with health care services?”, with five possible responses of: 1) Very satisfied; 2) Satisfied; 3) Medium; 4) Not satisfied; 5) Not at all satisfied.

Using the factors affecting user satisfaction with health services identified in the literature (**Table 1**), data was extracted from the LSS. Multivariate logistic regression analysis was used to control for the influencing individual characteristics available in the data. The health service satisfaction question shown above was changed to a binary ‘satisfied’ (combining 1 and 2 from the above)/ ‘unsatisfied’ (combining 3, 4 and 5 from the above) variable for ease of analysis, and used as the outcome measure in the regression model.

The independent variables included based on the available data were: year; age; gender; urban/rural; education; household income (socioeconomic status); services used in previous year (in order to assess relative satisfaction with type of service used); satisfaction with own health (as a proxy for self–assessed health status); and satisfaction with other services (as a proxy for psychological factors ie, general ‘satisfaction disposition’ of the individual).

The basic model being tested in the study is therefore:

y_i_ = α+β_1_X_1_+β_2_X_2_+β_3_X_3_+β_4_X_4_+β_5_X_5_ = β_6_X_6_+β_7_X_7_+β_8_X_8_+β_9_X_9_+ϵ_i_

where y_i_ = Satisfaction with health care services, α = Constant, X_1_ = Satisfaction with own health, X_2_ = Age, X_3_ = Gender, X_4_ = Urban/Rural, X_5_ = Educational level, X_6_ = Service used, X_7_ = Household income, X_8_ = Satisfaction with other services, X_9_ = Year, β_i_ = Coefficient, ϵ_i_ = Error.

The “satisfaction with other services” variable is a mean of binary satisfaction variables for satisfaction with: public security, criminal prosecution, education, social security, transportation, and general operations of public services, for each individual.

The regression model was run including only those who had used health services in the previous year (n = 43 143) in order to ensure the satisfaction measure matched to the year attributed to it.

## RESULTS

**Figure 1:**
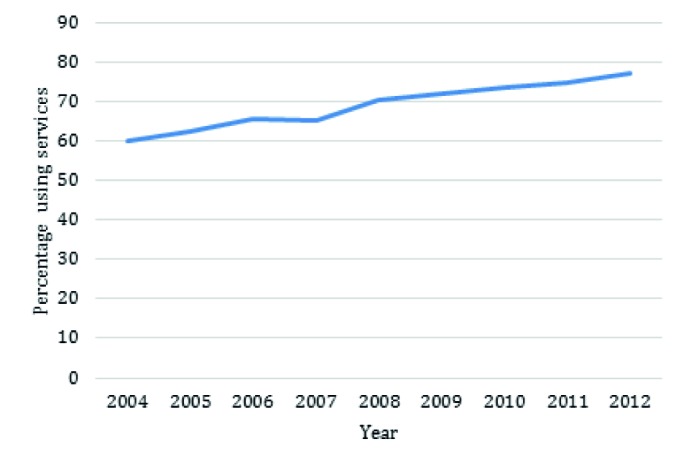
Percentage of survey respondents reporting having used any health services in the past year.

Over the period of the HTP reforms, a number of changes occurred in access to the health system, and where people chose to seek care. **Figure 1** shows increasing use of health services in general over the 9–year period, with an almost 20% rise in those reporting having used health services over the time period shown.

**Figure 2:**
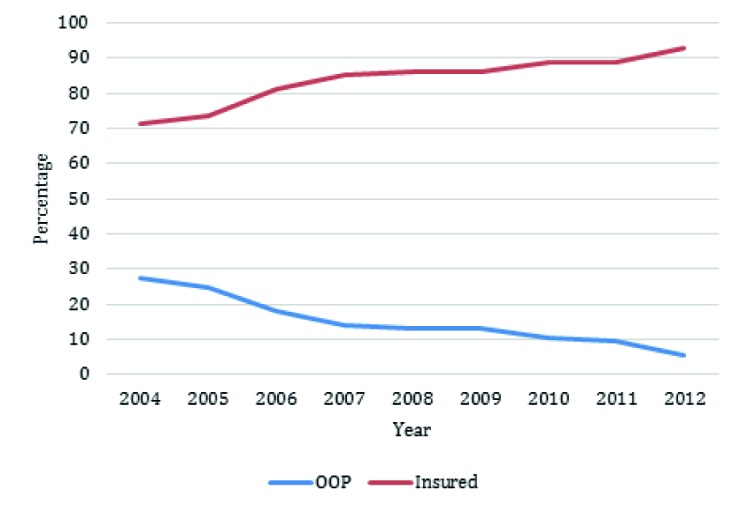
Percentage of respondents covered by health insurance or uninsured and paying out–of–pocket for health expenditures (by year, 2004–2012).

Increased access, shown by the insurance coverage over this same time period (**Figure 2**), is a likely contributor to this increased use.

**Figure 3:**
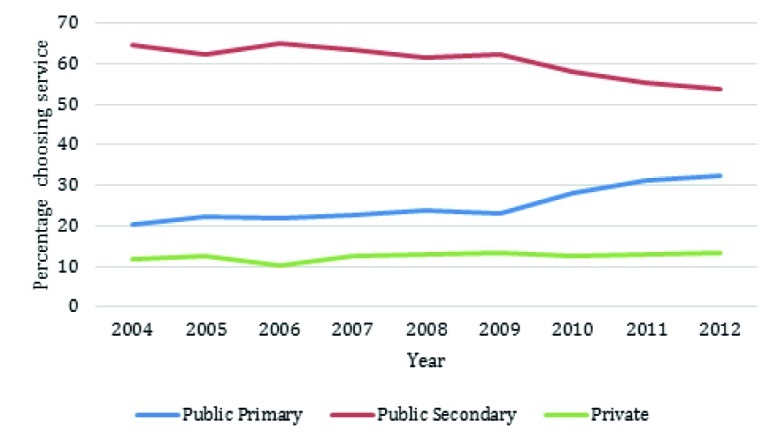
Proportion of those who would choose each service type for first–contact with health services.

Increased access and subsequent use of health services were accompanied with changes of providers where patients sought their first–contact with the health system. **Figure 3** shows the changes in choice of public or private health sector, and the proportion choosing public primary care services or public secondary care as their first point of contact.

Within this context of increased use of services and changing patterns of use of the different service types, we see changes in satisfaction with the health services being used.

**Figure 4:**
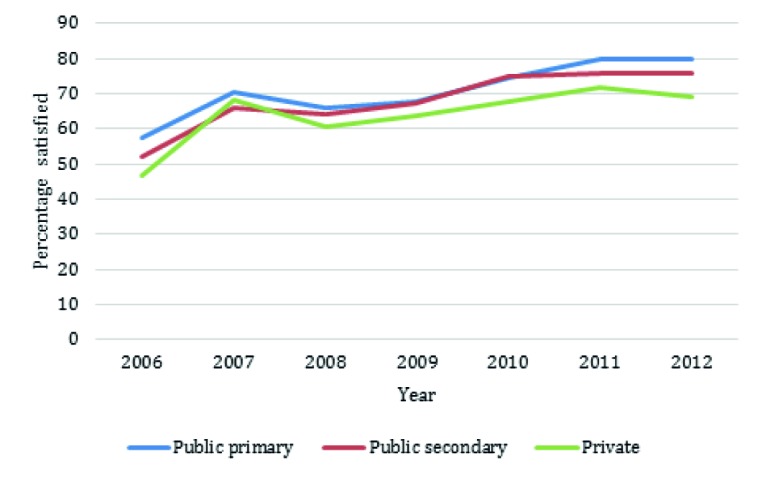
Satisfaction with health services by most recent service use and by year.

**Figure 4** shows trends for satisfaction levels. General satisfaction with all health services has improved steadily between 2006 and 2012, over the years of reforms, with the most rapid change occurring in the earliest years of data available for the ‘last use of service’ variable (between 2006 and 2007).

When the factors that affect user satisfaction with health services (**Table 1**) were controlled for using the multivariate regression model, the increases in satisfaction observed over the years holds true. Results from this model can be seen in **Table 3**. There was a significant (*P* < 0.001) increase in user satisfaction with health services in almost every year (bar 2006) from the baseline measure. In 2012, the odds of being satisfied with health services was 2.56 (95% confidence interval (CI) of 2.01–3.24) times that in 2004, having peaked at 3.58 (95% CI 2.82–4.55) times the baseline odds in 2011.

**Table 3:** Results of the multivariate logistic regression of satisfaction with health care services (n = 43 143 users of health services: 2004 to 2012 survey respondents)

	% in population	% satisfied with health services	Crude OR (95% CI)	Adjusted OR (95% CI)
**Satisfaction with own health:**				
Very satisfied/Satisfied	59.06	70.03	1	1
Medium	20.68	59.67	0.63 (0.60–0.67)†	0.56 (0.53–0.59)†
Not satisfied	16.97	57.71	0.58 (0.55–0.62)†	0.48 (0.45–0.51)†
Not at all satisfied	3.29	50.70	0.44 (0.40–0.49)†	0.35 (0.31–0.39)†
**Age:**				
18–34	32.99	58.92	0.83 (0.79–0.88)†	0.83 (0.79–0.88)†
35–49	30.55	63.23	1	1
50–64	22.81	69.64	1.33 (1.26–1.41)†	1.28 (1.20–1.37)†
65+	13.65	77.09	1.96 (1.82–2.10)†	1.86 (1.72–2.02)†
**Gender:**				
Male	38.87	64.31	1	1
Female	61.13	65.70	1.06 (1.02–1.11)†	1.14 (1.08–1.20)†
**Urban/Rural:**				
Urban	72.24	63.07	1	1
Rural	27.76	70.61	1.41 (1.34–1.47)†	1.19 (1.13–1.26)†
**Education:**				
Illiterate	12.67	70.94	1	1
Primary education	51.40	69.39	0.93 (0.87–0.99)*	0.79 (0.73–0.85)†
Secondary education	27.38	59.18	0.59 (0.55–0.64)†	0.52 (0.47–0.57)†
University education	8.55	50.31	0.41 (0.38–0.45)†	0.44 (0.39–0.50)†
**Use of services:**				
Other	19.97	51.20	1	1
Public primary	15.08	72.24	2.48 (2.32–2.66)†	1.26 (1.01–1.58)*
Public secondary	51.23	68.53	2.08 (1.97–2.18)†	1.15 (0.92–1.43)
Private care	13.73	65.13	1.78 (1.66–1.91)†	1.16 (0.93–1.46)
**Household income:**				
Lowest bracket	18.26	68.58	1	1
Lower middle bracket	24.34	68.90	1.02 (0.95–1.08)	0.93 (0.86–0.99)*
Middle bracket	21.30	66.06	0.89 (0.84–0.95)†	0.87 (0.81–0.94)†
Higher middle bracket	19.98	62.44	0.76 (0.71–0.81)†	0.88 (0.82–0.95)*
Highest bracket	16.12	57.83	0.63 (0.59–0.67)†	0.90 (0.82–0.98)*
**Satisfaction with other services:**				
Unsatisfied	49.25	47.94	1	1
Satisfied	50.75	81.88	4.91 (4.70–5.13)†	4.43 (4.23–4.64)†
**Year:**				
2004	9.17	46.66	1	1
2005	9.85	54.82	1.39 (1.27–1.51)†	1.74 (1.58–1.91)†
2006	9.63	52.00	1.24 (1.13–1.35)†	1.25 (0.99–1.58)
2007	9.64	66.88	2.31 (2.11–2.52)†	2.24 (1.77–2.84)†
2008	10.42	63.65	2.00 (1.83–2.18)†	2.05 (1.62–2.60)†
2009	12.51	66.59	2.28 (2.09–2.48)†	1.98 (1.56–2.50)†
2010	11.91	73.51	3.17 (2.90–3.46)†	2.83 (2.23–3.59)†
2011	12.67	75.86	3.59 (3.29–3.92)†	3.58 (2.82–4.55)†
2012	14.20	75.35	3.49 (3.21–3.81)†	2.56 (2.01–3.24)†

OR – odds ratio, CI – confidence interval

*Significant at *P* < 0.05.

†Significant at *P* < 0.001.

Trends in the adjusted odds ratios (ORs) of the other variables agree with findings from published literature (**Table 1**). Interestingly, the above results indicate that those who used public primary care services were slightly, but significantly (*P* < 0.05) more satisfied than those who used any other services.

**Figure 5:**
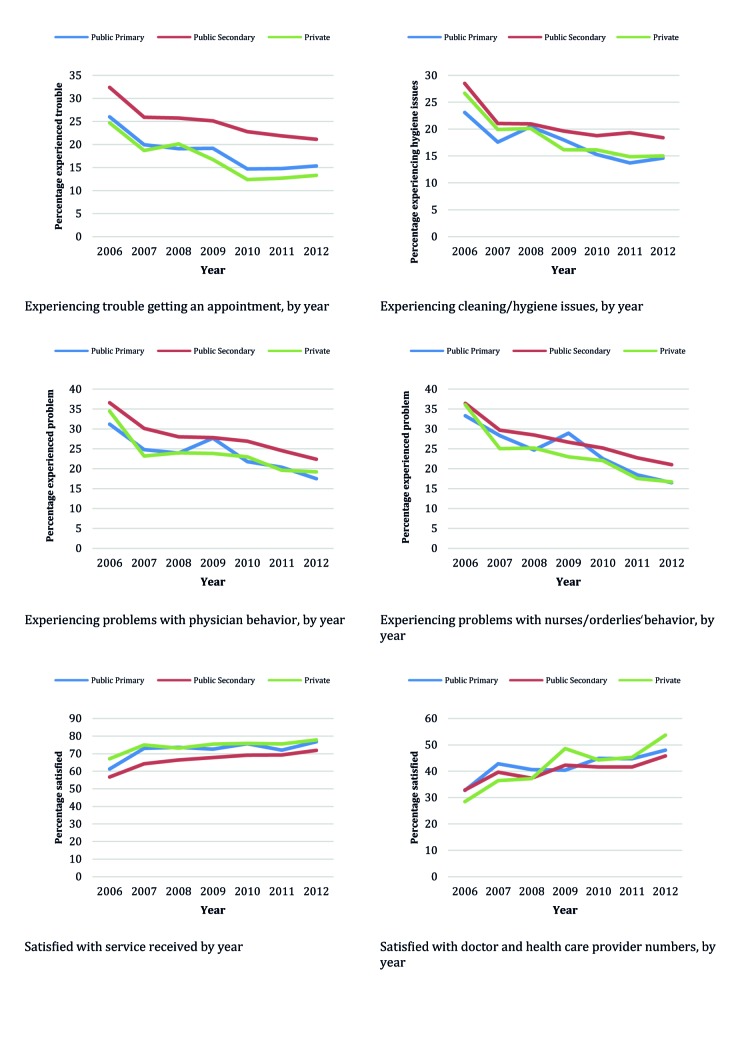
Experience of respondents in relation to quality factors.

To explain this increased satisfaction with primary care services, satisfaction with key aspects of service delivery were examined. **Figure 5** shows issues people had when using particular services. The quality of all services as perceived by the respondents appears to be improving over the years. Private and public primary care services appear to be the services people have the least problems in relation to perceived quality. These are also the services with which people are most satisfied with the providers.

These quality indicators can probably help account for the increased satisfaction with public primary care services, and the increase in seeking first–contact with these providers.

**Figure 6:**
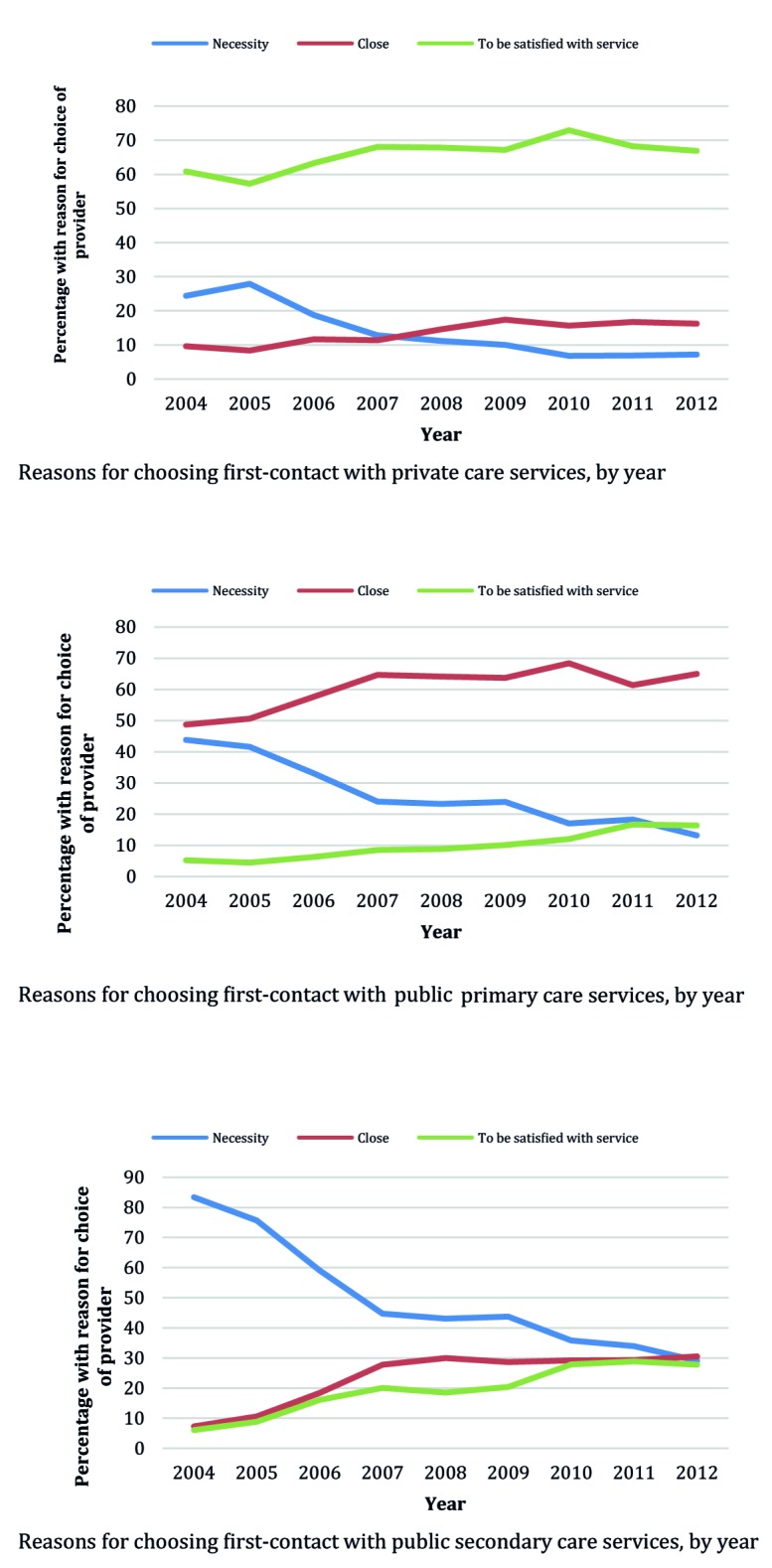
Reasons for choosing private sector, public primary care or public secondary care as the first point of contact provider.

The main reason identified by the respondents for choosing a private provider as the provider of first–contact service was satisfaction with the service, although the level of satisfaction remained around 60–65% between 2004 and 2012. Conversely, necessity as a reason declined over time from 25% to less than 10%, whereas proximity as a reason increased from 10% to almost 20% (**Figure 6**). The main reasons for choosing public primary care providers as the provider of first–contact service was closeness of the service, increasing from around 50% in 2004 to almost 70% in 2012. Necessity as a reason declined from more than 40% in 2004 to less than 10% in 2012. Conversely, satisfaction with services as the reason for choosing public primary care providers increased over time from around 5% in 2004 to almost 20% in 2012. While necessity was the main reason (more than 80%) for choosing a public secondary care provider in 2004, by 2012 this had declined to around 30%, while satisfaction with services and closeness of the services increased from less than 5% for both to around 30%.

The trends shown in **Figure 6** suggest that necessity as the main reason for choosing a specific service type is decreasing steadily. Respondents are increasingly choosing a particular type of service because they are satisfied with the service provided, particularly when choosing to use private care. Geographic accessibility as a reason is increasing for all services, reflecting the increasing availability and proximity of each type of provider as a result of the reforms and the ability of citizens to choose health care providers.

## DISCUSSION

The findings show that the user satisfaction with health services has increased significantly (*P* < 0.001) in Turkey over the period of HTP reforms, the implementation of which began in 2003, with scaling up of the new family medicine centered primary health care model from 2006 onwards. The statistically significant increase in user satisfaction levels holds after controlling for demographic factors, which also influence user satisfaction.

Similar directions of effect, as detailed in the earlier published literature, were found for each of the demographic factors analyzed. For example, those who were most satisfied with other public services (used as a proxy for psycho–social determinants) were much more likely (adjusted OR = 4.43 (95% 4.23–4.64)) to also be satisfied with health services. This measure is not commonly included in analyses of user satisfaction with health systems, but the large effect found in this study shows the importance of controlling for this factor in future studies when possible.

The rise in satisfaction levels is observed in the wake of large increases in overall use of health services over the period 2004–201), and the observed trend which suggests strongly that with the choice they have, the citizens are increasingly choosing primary care services rather than secondary care services as the provider of first contact.

The steepest increase in satisfaction can be seen early on in the reforms from 2006 following the nationwide implementation of the HTP. The early period of the reforms in 2004–2007 were the years when health insurance coverage for the poor citizens and access increased most rapidly, as seen in **Figure 2**. The elimination of costs for ambulance services, and threat of detention at a hospital with non–payment [5] would likely have also contributed to (at least perceived) accessibility of health services. Furthermore, the rapid increase in the scale and scope of services, which ensured nationwide expansion of provision of comprehensive services to cover the whole country, but especially the most needy citizens (through the Green Card scheme), and the incentives for deprived pregnant women and for children [1] through the conditional cash transfer schemes to use health services, would likely have influenced utilization and satisfaction levels.

Early in the health system reform, in 2004, HTP introduced for all citizens the right to directly choose health care providers in both the public and the private sectors, which had contracts with the Social Insurance Organization to provide health care services to those insured by the general health insurance scheme. With the rapid expansion of the new family medicine model, which was rolled out nationwide by 2010, the number of primary health care services available for citizens to choose increased. Earlier studies suggest that increases in access to and use of primary care services are associated with a rise in user satisfaction levels [28]. Similarly, having a choice of provider is also associated with increased satisfaction with health services [16,23].

**Figure 6** highlights the reasons for the increased satisfaction with primary care services, where patients report fewer problems with health service quality and report greater levels of satisfaction with the health services received.

We report data from 2006 to 2012, as data on specific health service use were not collected until then, limiting the period of analysis possible, but the period of analysis coincides with the scale up of family medicine centered primary care services. The lack of a regional identifier at province level has limited our ability to specifically analyze the effects of PHC on user satisfaction as the FM model was gradually rolled out across the country, but we were able to use the nationally representative annual survey data to ascertain effects of the national expansion of the FM model (**Box 1**).

The main aims of HTP were to extend health insurance to all citizens through government financing of the Green Card scheme, and by consolidating the five parallel insurance/financing schemes into a unified general health insurance, expanding access to health services, especially to primary health care, and thereby promote UHC. In addition, HTP also introduced for the citizens of Turkey the choice of health care providers, thereby improving the responsiveness of the health system to the users. Collectively, these changes, briefly summarized in **Box 1**, contributed to increased user satisfaction with the health system. The government regularly used the Life Satisfaction Survey to assess the perceptions of the citizens of the health system reforms and to fine–tune the reforms so as to improve the responsiveness of the health system to users and meet their expectations [1]. This ongoing learning is an important lesson for future health system reforms in Turkey and for countries undertaking health system reforms to achieve UHC. Nationally representative, consistent and rigorous surveys of user satisfaction at the start of and through implementation of health system reforms is very rare, and has been found lacking in most health system reforms [17,32]. Annual surveys of user perceptions of health system reforms using a nationally representative sample in Turkey provides an example of good practice that incorporates assessment and evaluation tools to provide evidence and inform implementation of reforms.
